# Neuroendocrine Differentiated Breast Cancer Cases: A Retrospective Analysis and Literature Review

**DOI:** 10.14744/SEMB.2021.66503

**Published:** 2021-12-29

**Authors:** Ozlem Ozdemir, Baha Zengel, Yasar Yildiz, Seray Saray, Ahmet Alacacioglu, Funda Tasli, Zuleyha Can Erdi, Utku Oflazoglu, Halil Taskaynatan, Tarik Salman, Umut Varol, Zehra Hilal Adibelli, Raika Durusoy, Yuksel Kucukzeybek

**Affiliations:** 1. Department of Medical Oncology, University of Health Sciences, Izmir Bozyaka Training and Research Hospital, Izmir, Turkey; 2. Department of General Surgery, University of Health Sciences, Izmir Bozyaka Training and Research Hospital, Izmir, Turkey; 3. Department of Medical Oncology, Izmir Katip Celebi University, Ataturk Training and Research Hospital, Izmir, Turkey; 4. Department of Medical Pathology, University of Health Sciences, Izmir Bozyaka Training and Research Hospital, Izmir, Turkey; 5. Department of Medical Internal Medicine, University of Health Sciences, Izmir Bozyaka Training and Research Hospital, Izmir, Turkey; 6. Department of Radiology, University of Health Sciences, Izmir Bozyaka Training and Research Hospital, Izmir, Turkey; 7. Department of Public Heath, Ege University, Izmir, Turkey

**Keywords:** Mammography, Neuroendocrine breast carcinoma, Neuroendocrine differentiation

## Abstract

**Objectives:**

Neuroendocrine breast carcinoma (NEBC) is a rare subgroup of breast cancer, which makes up 2–5% of all invasive breast cancers. The aim of this retrospective analysis is to present and analyze our own data of primary NEBCs.

**Methods:**

We retrospectively analyzed clinical, pathological, and radiological characteristics of 36 patients diagnosed with neuroendocrine differentiated breast cancer between 2008 and 2019 compared to that of 925 patients with invasive ductal carcinoma (IDC/NOS) along with a literature review.

**Results:**

In this study, 36 patients with neuroendocrine differentiated breast carcinoma and 961 patients with (IDC/NOS), as the comparison group, were identified between 2008 and 2019. In NEBC patients, seven were premenopausal and 29 postmenopausal. Patients whose ultrasound (USG), magnetic resonance, and mammographic (MMG) images available in our hospital, high-density masses were detected in the MMG with irregular (77%), microlobulated (80%) and spiculated margins (63%), unaccompanied by asymmetry and structural distortion. Calcifications were less common than invasive breast cancer, present only in four patients (17%). When NEBC were compared to ductal carcinomas (n=925), NEBC were more often human epidermal growth factor receptor 2 negative (p=0.039), estrogen receptor positive (p=0.05), progesterone receptor positive (0.03), and the NEBC patients were older (p=0.02). Age, grade, metastatic status, lymph node number, and molecular type were identified as prognostic factors that significantly affect survival in both groups (p<0.05).

**Conclusion:**

NEBC is a subtype that is both histopathologically and radiologically distinct from other breast cancer subtypes, and neuroendocrine differentiation may be an important predictive marker in the future.

Breast cancer is the second most common type of cancer worldwide. The most common type of breast cancer is invasive ductal carcinoma (IDC). Neuroendocrine neoplasia (NEN) is a rare, heterogeneous tumor group with variable clinical behavior due to the differentiation of the tumor, which originates from the endocrine system. It is reported that the primary neuroendocrine tumors of the breast are caused by early-stage variable differentiation of breast cancer stem cells. Stem cells are considered to differentiate into both neuroendocrine and epithelial lines as a result of this phenomenon. Primary neuroendocrine tumor of the breast is generally diagnosed by the microscopic detection of neuroendocrine structure in the cancer cell and the presence of neuroendocrine markers such as chromogranin A and synaptophysin. Neurone-specific enolase (NSE) and CD56 may also be positive, but they are not as sensitive and specific as the former two.^[1]^ The 2003 World Health Organization classification of breast cancers stated that neuroendocrine markers being more than 50% were adequate for diagnosis. In the 2012 version, this threshold was omitted, and expressing neuroendocrine markers were considered adequate. In the 2012 classification, three types of tumors were defined: well-differentiated neuroendocrine breast carcinoma (NEBC) (NETs, which included low- and intermediate-grade tumors), poorly differentiated NEBC/small cell carcinoma, and NEBC determined by histochemistry or immunohistochemistry(IHC).^[2]^ Briefly, in 2012, the WHO classified primary NET and NECs together with grade 1 or 2 breast carcinomas that express neuroendocrine indicators in a single group under the heading “Breast carcinoma with neuroendocrine differentiation.” However, in 2019, the WHO recommended a grouping similar to the neuroendocrine carcinoma classification in other organs. In the 2019 WHO classification, the primary neuroendocrine tumors of breast were defined in three categories as neuroendocrine tumors (NET), large cell neuroendocrine carcinoma and small cell neuroendocrine carcinoma. These breast tumors are sporadic subtypes, and their common characteristics are uniform neuroendocrine morphology, presence of neurosecretory granules in cells, and diffuse-uniform neuroendocrine marker staining. 2019 WHO classification recommended defining the tumors with invasive breast carcinoma having nonspecific and specific morphology types (mucinous carcinoma, solid papillary carcinoma, etc.) and non-uniform neuroendocrine morphology and neuroendocrine marker expression as “Invasive carcinomas of the breast with neuroendocrine differentiation.”^[2]^ In very few cases, the secretion of hormones such as ACTH, norepinephrine, or calcitonin and associated findings were observed.^[3]^ NENs can occur in almost all organ systems. In most cases, NEN occurs within the gastroenteropancreatic system (70% of all cases) and the bronchopulmonary system (25%).^[4]^ Mammary origin accounts for less than 1% of neuroendocrine tumors.^[5]^ Breast cancer incidence rates are reported to vary between 0.1% and 5%, and these tumors are thought to arise from endocrine differentiation of breast carcinoma rather than from pre-existing endocrine cells with malignant transformation.^[6]^ Because it is a rare breast carcinoma, there are limited studies on its prognosis with conflicting results. In an epidemiological study.^[7]^ In a compilation of 53 articles, including 108 cases in total, Adams et al. showed that prognosis is usually quite favorable with small tumors and no nodal involvement.^[8]^ There is still no consensus on the effect of NEBC on prognosis. In the present study, we retrospectively analyzed the clinicopathological characteristics of NEBC and breast carcinoma.

## Methods

In this study, among the patients diagnosed with breast cancer in in Bozyaka Training and Research Hospital (TRH) and Katip Çelebi University Atatürk TRH oncology outpatient clinics between 2008 and 2019, 36 patients with neuroendocrine differentiated breast carcinoma and 925 patients with IDC (IDC/NOS), as the comparison group, were identified. Information on demographic data (including name, gender, age, and contact information), physical examination, radiological findings, surgical procedures, histopathological and immunohistochemical characteristics, systemic adjuvant/neoadjuvant therapy, and follow-up were retrospectively collected. Before the study, approval was obtained from the clinical research ethics committee of our hospital on (decision date 17.09.2020 and number: 05), and there is no conflict of interest between the authors. The American Joint Committee on Cancer (AJCC) TNM was used for staging. The estrogen receptor (ER), progesterone receptor (PR), human epidermal growth factor receptor 2 (HER-2), Ki-67 proliferation index, and oncoprotein P53, which are associated with breast cancer, were immunohistochemically evaluated. At least 1% of tumor cells being stained were considered ER and PR positive, and immunohistochemical staining 3+ was considered HER-2 positive. On the other hand, in cases with immunohistochemical HER-2 +2, fluorescent in situ hybridization was checked. For cases in the study, the threshold value for Ki67 immunochemical staining was taken as 14%.^[12]^ Tumors with a profile of ER and/or PgR (+)/HER2(–)/Ki67 ≤14% were classified as Luminal A, ER and/or PgR (+)/HER2 (+) or (–)/Ki67 >14% tumors Luminal B, ER(–)/PgR(–)/HER2 (+) tumors HER2 overexpressed, and ER(–)/PgR(–)/HER2(–) Triple-negative breast cancer. Besides, the expression of neuroendocrine markers with immunohistochemical staining was assessed, including chromogranin A (CGA), synaptophysin (SYN), NSE, and CD56. In immunohistochemical staining, the presence of at least one non-uniform expression of CGA or SYN was considered neuroendocrine marker-positive. In the survival analysis of patients, disease-free survival (DFS) was assessed by calculating the time from diagnosis to relapse and overall (OS) survival from diagnosis to death.

Statistical analysis was performed using IBM SPSS Statistics Software. Descriptive statistics were calculated for demographic and clinicopathological factors. Differences in these factors between NEBC and IDC, NOS of the breast were compared using Chi-square or Fisher exact test, where appropriate, for categorical variables and using the Student t-test to compare means. DFS, survival were calculated from the time of diagnosis to disease recurrence at any site (DFS), overall (OS) survival were calculated from the time of diagnosis to death from any reason (OS). Survival curves were constructed using the Kaplan–Meier method, and differences between curves were analyzed using the Log-rank test. Multivariate analysis for survival time was performed using the Cox proportional hazards model. In statistical analysis, p<0.05 was considered significant.

## Results

All the patients included in the present study were female and presented with palpable breast mass at a rate of 82%. Regarding the age of diagnosis, the median age was 59 (min: 33, max: 86) in cases with neuroendocrine differentiated breast carcinoma and 51 (min: 23, max: 93) in the comparison group IDC/NOS (n=925). The difference between them was statistically significant (p=0.02). The mean tumor size was 31 mm in the NECB group and 28 mm in the comparison group, which was not statistically significant (p>0.05). According to the AJCC (pTNM) staging system, in the NECB group, the number of stage I patients was 6 (16.6%), stage II 14 (38.8%), stage III 11 (30.5%), and stage IV 5 (13.6%). In the IDC/NOS group, the number of stage I patients was 173 (18.7%), stage II 469 (50.7%), stage III 255 (27.5%), and stage IV 28 (3%). The difference between the two groups for all stages was statistically significant (p=0.005). In NEBC patients, 7 were premenopausal and 29 postmenopausal (Table 1), and in the comparison group, 485 patients were premenopausal and 440 postmenopausal. The difference between the groups was statistically significant (p<0.001). Regarding the magnetic resonance imaging (MRI) of patients, in the NECB group, 2 were multicentric, 11 multifocal, and 23 unifocal. In the comparison group, 41 were multicentric, 132 multifocal, and 752 unifocal. The difference between the groups was not statistically significant (p>0.05). Besides, out of the 36 cases with NECB, in 23 patients with ultrasound (USG), magnetic resonance (MR), and mammographic (MMG) images available in our hospital, high-density masses were detected in the MMG with irregular (77%), microlobulated (80%) and spiculated margins (63%), unaccompanied by asymmetry, and structural distortion. Calcifications were less common than invasive breast cancer, present only in 4 patients (17%). In USG, common findings were hypoechoic (68%), borderless (63%), and no posterior acoustic features (59%). The MRI showed masses with irregular (100%), microlobulated (54%) and spiculated (27%) margins, and isointensity to parenchyma on T1 (100%). Regarding the molecular subtypes of NECB patients, 16 were Luminal A, 13 Luminal B HER2(–), and 7 Luminal B HER 2(+). 7 patients (19.4%) were HER-2+ in the NECB group. NEBC were compared to ductal carcinomas (n=925), NEBC were more often HER2 negative (p=0.039), ER positive (p=0.05), PR positive (0.03). The mean follow-up time was 66 months in the NEBC group and 107 months in the IDC/NOS group. The difference between the groups was significant (p<0.001). Two of 31 non-metastatic patients received neoadjuvant therapy and 29 adjuvant therapy. Four (11.2%) of patients with NECB follow up, and 252 (27.2%) of IDC/NOS patients died. The difference was statistically significant (p=0.034). However, there was no significant difference between the two groups in terms of histological grade, e-cadherin, vascular invasion, neural invasion, relapse, metastasis, or DFS. Survival analysis excluding the number of metastatic patients also revealed no difference between the groups in terms of DFS and OS. Age, grade, metastatic status, lymph node number, and molecular type were identified as prognostic factors that significantly affect survival in both groups (p<0.05).

**Table 1.** Clinicopathological characteristics of neuroendocrine carcinomas and IDC at the time of diagnosis

Mean age	59.0
Menopausal status	
Premenopause	7 (19.4%)
Postmenopause	29 (80.5%)
T Stage	
T1	11 (30.5%)
T2	18 (50%)
T3	6 (16.6%)
T4	1 (2.7%)
N Stage	
N0	12 (33.3%)
N1	14 (38.8%)
N2	7 (19.4%)
N3	3 (8.3%)
Diagnosis stage	
Stage I	6 (16.6%)
Stage II	14 (38.8%)
Stage III	11 (30.5%)
Stage IV	5 (13.8%)
Molecular type	
Luminal A	16 (44.4%)
Luminal B HER2(–)	13 (36.1%)
Luminal B HER2(+)	7 (19.4%)
Grade
Grade 1	3 (8.3%)
Grade 2	25 (69%)
Grade 3	8 (22%)
Synaptophysin expression	
Yes	33 (91.6%)
No	3 (8.3%)
Chromogranin expression	
Yes	21 (58.3%)
No	15(41.6%)
MR	
Multicentric	2 (5.5%)
Multifocal	11 (30.5%)
Unifocal	23 (63.8%)
Neoadjuvant chemotherapy	
Yes	2 (5.5%)
No	34 (94.5%)
Adjuvant chemotherapy	
Yes	29 (80.5%)
No	7 (19.4%)

## Discussion

The neuroendocrine tumor of the breast was first described in 1963 by Feyrter and Hartmann.^[9]^ In 1977, Cubilla and Woodruff presented the first case series and identified breast cancers with neuroendocrine differentiation as primary carcinomas of breast.^[10]^ The histogenesis of the tumor has not been fully clarified yet and is often thought to result from the differentiation of multipotent stem cells into the neuroendocrine carcinoma phenotype.^[11]^ The diagnosis of neuroendocrine tumor of the breast is generally established by microscopic detection of the neuroendocrine structure in the cancer cell and the positive neuroendocrine markers such as CGA and SYN. NSE and CD56 can also be checked, but they are not as sensitive and specific as the former two.^[1]^ In the 2003 classification, the prevalence of these tumors was estimated to be between 2% and 5%. However, in the Surveillance Epidemiology and End Results database, only 142 NEBC cases were identified between 2003 and 2009, which corresponds to a prevalence of <0.1%.^[12]^ In sum, a full literature review shows that the incidence of neuroendocrine breast tumors has been reported in rates ranging between 0.3% and 20%. In our case series, this rate was 3%, and the lack of uniform morphological and immunohistochemical diagnostic criteria could account for the different prevalence results reported in the literature. In most cases, NECB appears as a painless palpable retroareolar mass with secondary symptoms such as skin ulcers, bloody nipple discharge, lymphadenopathy, or retraction of the nipple.^[6]^ All of our patients had presented to our clinic for palpable mass. Most NECB patients are postmenopausal women of advanced age, and the incidence of the disease is lower in men and younger women.^[6]^ In the literature, the age of onset has been reported as most frequent in the 6th and 7^th^ decades. Hence, the mean age 61.1 in the present study is consistent with literature. In NEBC patients, 7 were premenopausal and 29 postmenopausal, and in the comparison group, 485 patients were premenopausal and 440 postmenopausal. The difference between the groups was statistically significant (p<0.001). Radiological features of NECB are nonspecific in most cases. Some investigators have stated that NEBCs are observed as hypoechoic masses with irregular morphology in mammography and USG, as well as small-sized lesions not associated with microcalcifications.^[13]^ In the present study, high-density masses were detected in the MMG with irregular (77%), microlobulated (80%) and spiculated margins (63%), unaccompanied by asymmetry and structural distortion, along with microcalcification in 4 patients (17%). It may also appear in USG as hypervascular, hypoechoic solid masses, with irregular form and enhanced posterior echogenicity.^[13]^ In our study, hypoechoic (68%), borderless (63%), no posterior acoustic characteristics (59%) were common findings in the USG. The most frequent findings in MR are irregular masses with irregular margins and wash-out time-intensity kinetics, which are features highly suspicious for malignancy.^[14]^ Some studies also noted that irregular lesions are often detected by hypointense on NECB-T1 weighted sequences with early and intense enhancement.^[13]^ In our study, the MRI showed masses with irregular (100%), microlobulated (54%) and spiculated (27%) margins, and isointensity to parenchyma on T1 (100%). Fine needle aspiration cytology is not recommended due to the similarity of NECB’s cytological features to IDC and intraductal papilloma.^[1]^ The use of detailed immunohistochemical staining and various imaging techniques is essential for an accurate diagnosis. CgA and SYN are the most sensitive neuroendocrine markers,^[15]^ whereas NSE and CD56 are less sensitive and less specific.^[16]^ While NECB is usually positive for hormone receptors (ER, PR), HER2 is almost always negative, although there have been reports of HER2(+) NECB.^[10]^ In the present study, regarding the molecular subtypes of the 36 NEBC patients, 16 were Luminal A, 13 Luminal B HER2(–), and 7 Luminal B HER2(+). 7 patients (19.4%) were HER-2(+) in the NECB group. When NEBC were compared to ductal carcinomas (n=925), NEBC were more often HER2 negative (p=0.039), ER positive (p=0.05), PR positive (0.03).

Due to the similarity to neuroendocrine tumors, the NECB can easily be confused with metastases of neuroendocrine tumors to the breast. The presence of a ductal in situ component is histological evidence that the breast is the primary organ of origin.^[17]^ Besides, an expression of transcription factors such as GATA3, a more sensitive and specific marker than mammaglobin, also indicates breast origin. In addition, positive expression of hormone receptors (PR, ER) in well/moderately differentiated NECB is instrumental in differentiating primary and secondary lesions.^[18]^ In the present study, the expression neuroendocrine markers were investigated with immunohistochemical staining, including CGA, SYN, NSE, and CD56. In immunohistochemical staining, the presence of at least 1 non-uniform expression of CGA or SYN was considered neuroendocrine marker-positive. The prognostic implications of neuroendocrine differentiation in breast carcinoma are still debated. Historically, based on small-scale studies, NEBC was thought to have a prognosis that is similar,^[19]^ or even better,^[20]^ compared to IDC. However, recent studies have suggested that NEBC could be associated with worse long-term outcomes.^[6]^ In addition, a large retrospective study by Zhang et al. reported a higher probability of local recurrence and poorer OS for NEBCs.^[21]^ In the available literature, the prognostic factors affecting survival are indicated as disease stage, number of lymph-node metastases, and ER and PR status.^[22]^ In the present study, age, grade, metastatic status, number of lymph nodes and molecular type were identified as prognostic factors that significantly affect survival in both groups. The limited number of studies in the literature and lack of standardization in definition and classification may account for these conflicting results concerning the clinical outcome of NEBC. Similarly, some authors have investigated the possible effect of histological subtyping of NEBC according to the 2012 WHO classification on prognosis, providing different evidence. Cloyd et al. have shown that small cell carcinoma subtype is associated with worse DSS and OS compared to well-differentiated NECB and invasive carcinoma with neuroendocrine characteristics.^[23]^ Four (11.2%) the patients with NECB follow-up, and 252 (26.4%) the IDC/NOS patients died. The difference was statistically significant (p=0.034). However, the difference in terms of survival was not significant between the groups. The absence of a significant sign or symptom and the lack of standards in diagnosis bring about challenges in diagnosing NECB. Similar to typical luminal subtypes of breast cancer, NEBC can metastasize to multiple sites, the most frequent being bone and liver.^[24]^ In terms of stage IV disease, there was metastasis in 5 patients (13.9%) in the NECB group and 38 (4.1%) in the comparison group. The difference was statistically significant (p=0.005). However, there was no significant difference with regard to location of metastasis. Treatment is similar to that for other conventional types of invasive breast carcinomas and depends on tumor size, location, and clinical stage.^[25]^ There is a general consensus on treating small cell NECB with chemotherapy regiments similar to these for small cell lung carcinoma.^[26]^ While most NECB treatments reported in the literature (regarding well/moderately differentiated NECB) are similar to ductal type treatment, Anlauf et al. emphasize the importance of treatment according to NET guidelines.^[27]^ Up to date, there is not sufficient data to determine the most effective chemotherapy regimen. In general, poorly differentiated or small cell NEBCs are treated with regimens containing platinum/etoposide, whereas anthracyclines and/or taxanes-based chemotherapy are used for other types of NEBCs.^[28]^ The effectiveness of antihormonal treatment has been demonstrated in patients with hormone receptor-positive breast carcinoma. Richter-Ehrenstein et al. suggested using adjuvant antihormonal therapy as the standard treatment approach in the hormone receptor-positive NECB.^[29]^ The prognostic role of HER-2 in NECB is not clear, but it can be assumed that it is analogous to other invasive breast carcinomas, meaning that anti-HER2 therapy is recommended for HER2-positive NECB. Somatostatin receptors are G-protein-coupled receptors expressed by NET cells at lung, prostate and gastrointestinal level, as well as by ductal breast cancer cells. In SSR-positive tumors, peptide receptor radionuclide treatment (PRRT), a tumor-targeted systemic radiotherapy, has been recommended as a treatment alternative in unresponsive cases.^[30]^ In the present study, no somatostatin analogues or PRRT were administered in the treatment of patients, and they have been treated like invasive breast carcinoma. As a result, many studies have shown that these adjuvant systemic treatments, such as chemotherapy, radiotherapy and endocrine therapy, can play an important role and should be administered according to the individual characteristics of NEBC patients.^[16,31]^

## Conclusion

Primary neuroendocrine carcinoma of the breast is a rare tumor, classified as a subtype of invasive breast carcinoma with particular histopathological features. Recent developments in oncology and targeted treatment plans have shown that molecular biology of tumor cells is crucial. However, there is no consensus on the prognosis of neuroendocrine tumors of the breast compared to other breast tumors. They are treated like invasive breast carcinoma due to their rarity, the absence of randomized data, and limited evidence to guide treatment selection. Despite the lack of significant survival data due to the limited number of patients in the present study, we think NEBC is a subtype that is both histopathologically and radiologically distinct from other breast cancer subtypes, and neuroendocrine differentiation will become more predictive in the future.

### Disclosures

**Ethics Committee Approval:** Before the study, approval was obtained from the clinical research ethics committee of our hospital on (decision date 17.09.2020 and number: 05), and there is no conflict of interest between the authors.

**Peer-review:** Externally peer-reviewed.

**Conflict of Interest:** None declared.

**Authorship Contributions:** Concept – O.O., Y.Y., A.A., Z.H.A.; Design – O.O., S.S., F.T., Z.C.E.; Supervision – O.O., B.Z., U.O., H.T., T.S.; Materials – O.O., B.Z., U.V., U.O., Z.C.E., S.S.; Data collection &/or processing – O.O., S.S., Y.K., A.A.; Analysis and/or interpretation – O.O., R.D., A.A., T.S., Y.K.; Literature search – O.O., B.Z., Y.Y., U.O.; Writing – O.O., A.A., Y.K.; Critical review – O.O., A.A., Y.K., F.T.
